# Potential Value of Qiagen and PrepIT•MAX Kits in Extraction of Mycobacterial DNA From Presumptive Tuberculosis Archived Formalin-Fixed Paraffin-Embedded Tissues

**DOI:** 10.24248/EAHRJ-D-17-00256

**Published:** 2018-04-01

**Authors:** Yunus Ayub, Jackson T Mollel, Erasto V Mbugi

**Affiliations:** a Biochemistry Department, School of Medicine, Muhimbili University of Health and Allied Sciences, Dar es Salaam, Tanzania; b Department of Biological and Pre-Clinical studies, Institute of Traditional Medicine, Muhimbili University of Health and Allied Sciences, Dar es Salaam, Tanzania; c Ministry of Health, Community Development, Gender, Elders & Children, Department of Human Resources Development, Singida Health Laboratory Assistants Training Centre, Singida, Tanzania

## Abstract

**Background:**

DNA analysis has potential for screening for and diagnosing a variety of conditions as well as the characterization of various pathogens for many purposes including to identify genetic disorders and mutations, study genetic diversity, and establish evolutional trends.

**Methods:**

Our study compared the performance of 2 DNA extraction kits: Qiagen and prepIT•MAX. The study tested 160 formalin-fixed paraffin-embedded (FFPE) human tissue samples that had been collected at Muhimbili National Hospital (MNH) between 2010 and 2016. For each sample, DNA extraction was performed using both the Qiagen and prepIT•MAX kits followed by polymerase chain reaction (PCR) tests to target the RNA polymerase gene and gel electrophoresis.

**Results:**

The findings showed that the Qiagen was 3 times superior to the prepIT•MAX kit in successfully extracting mycobacterial DNA from presumptive tuberculosis (TB) FFPE tissues. Of the 160 previously Ziehl-Neelsen stain-negative *Mycobacterium tuberculosis* suspicious tissue samples, 12 (7.5%) tested positive with the PCR. Of the 12 PCR-detected positive samples, 8 (66.7%) yielded positive results with the Qiagen kit only and 4 (33.3%) yielded positive results with both Qiagen and prepIT•MAX kits. Additionally, 10 (83.3%) came from well-formed granuloma, 1 (8%) from caseous necrosis, and 1 (8.3%) Langhans-type giant cells endorsing their potential for housing infection such as TB adenitis.

**Conclusions:**

A combination of molecular techniques, microscopy, and pathological features increases detection of *M. tuberculosis* from FFPE tissues. Both the Qiagen and the prepIT•MAX DNA extraction kits have shown a remarkable capability for extracting DNA from *M. tuberculosis*, although examination of FFPE tissues is not an intended use for the prepIT MAX, according to the manufacturer. In resource-limited countries, however, these kits may complement each other. We recommend further studies for validation and optimization, which includes the cost effectiveness of prepIT•MAX extraction kit to advocate for its use in extraction of mycobacterial DNA from FFPE tissues.

## INTRODUCTION

With advances in technology, archived biological materials have increasingly become important sources of scientific data. Modern technology can enable, for example, a detailed investigation and analysis of preserved tissue samples at molecular level. As a result, it is possible to retrospectively and prospectively explore large numbers of patients and detect and track genetic changes in infectious agents that occur over time.^1,2^ Consequently, this method of analysis provides room for planning appropriate remedies to various disease conditions.

Formalin-fixed paraffin-embedded (FFPE) human tissues have been suitable specimens as DNA sources. DNA analysis is simple, extremely sensitive, and uses small sample amounts (5 to 10 μm sections) for extraction. This method also allows analysis of DNA from a large number of archived tissue samples within short time.^3,4^ Nevertheless, for a successful outcome, this approach requires that an appropriate DNA extraction method is used to produce quality DNA data.^5^ This is particularly important when analysing various tissue samples from different sources and different storage systems. Archived tissue samples enable analysis of gene expression^6,7^ and proteome analysis^8,9^ even in preserved tissues where the collection procedures are largely unknown. FFPE tissues that have been stored for years can provide an opportunity to study macromolecules like DNA, RNA, and proteins for valuable information with no significant difference over storage time.^10^

In extrapulmonary tuberculosis (TB),*Mycobacterium tuberculosis* infects cells in tissues and organs outside the lung. In latent extrapulmonary TB, infected tissues/organs act as reservoirs for reactivation of active TB.^11^ This is particularly important in zoonotic TB in multispecies host–pathogen ecosystems where livestock, livestock products, and people co-exist and, thus, are at high risk of infection.^12^ In such situations, an innovative, time-efficient, affordable, and applicable-to-field-conditions approach is important for disease surveillance, particularly in low-income countries.

Molecular tools are important for the management, surveillance, and control of diseases. They require appropriate tissue samples that were preserved in ideal conditions and suitable DNA extraction evaluation tools. To obtain DNA for analysis, proper choice of preferential sites where one is most likely to get DNA samples may be necessary to achieve expected outcomes. Detection of *M. tuberculosis* complex in FFPE tissue specimens with necrotizing granulomatous inflammation provides rapid and correct diagnosis on different sources of preserved samples collected prospectively. In so doing, this method offers supplementary opportunity for TB diagnosis in patients where TB was not initially suspected.^13^ Polymerase chain reaction (PCR) testing has long been recommended for detection of *M. tuberculosis* DNA in FFPE specimens; it offers increased sensitivity and accuracy crucial for patients with perplexing diagnostic problems associated with a granulomatous tissue response^14^ and unusual presentation.^15^ Other studies have recommended use of PCR when TB is suspected clinically, especially in cases of chronic inflammation without definite evidence of granulomatous inflammation.^16^ Heating the tissue under the influence of variable pH values has been shown to be an effective protocol for DNA extraction from archived paraffin-embedded tissues and may contribute in providing enhanced understanding of changes that occur during formalin-induced modification of nucleic acids.^17^ Our study aimed at using FFPE archived human tissue collected retrospectively for diagnosis of *M. tuberculosis* in presumptive TB patients. We compared the performance of 2 commercial kits – Qiagen and prepIT•MAX – to extract DNA from archived FFPE tissue samples, after which each of the samples was tested by PCR amplification of the RNA polymerase (*rpo*B) gene and detection by gel electrophoresis.

## METHODOLOGY

### Study Design and Population

This retrospective cross-sectional study involved 160 archived FFPE samples that had been collected as part of routine disease management at Muhimbili National Hospital (MNH) central pathology laboratory from 2010 to 2016. The study included tissues that were noted to have histopathological features suggestive of non-specific chronic inflammation and granulomatous lesions. These samples were tested to find out whether they originated from presumptive TB cases. Presumptive TB refers to a patient who presents with symptoms or signs suggestive of TB, previously known as a TB suspect. DNA extraction and PCR analysis from selected samples was done at the Molecular Biology Laboratory, Department of Biochemistry at Muhimbili University of Health and Allied Sciences (MUHAS).

### Inclusion and Exclusion Criteria

The inclusion criteria involved all reported cases presumed of non-specific chronic inflammation and granulomatous lesions as detected by haematoxylin and eosin (H/E) staining. All reported cases with benign or malignant features and tissues with normal histological patterns, as detected by H/E staining, were excluded from the study. Archived types of tissues initially submitted for evaluation for other granulomatous inflammatory conditions were tested by the Ziehl-Neelsen (ZN) technique and then DNA extraction by the 2 commercial kits (Qiagen and prepIT•MAX) to establish potential for extrapulmonary TB. Biopsies of individuals submitted for histopathological evaluation were selected using simple random sampling (computer-generated table of random numbers) after obtaining patients' medical records from the archived files and log books. The sample size was then adjusted using the finite population correlation factor to arrive to 160 tissue samples from the calculated 207 sample size. The obtained number of tissue samples was considered enough to provide sufficient information to draw useful conclusions about the compared techniques.

### Data Collection and Quality Control

For clinical and social-demographic data, a form was developed to capture information including age, gender, area of residence, medical history, HIV status, methods used for their diagnosis, site of the specimen, and final diagnosis. All necessary safety requirements for the investigator were observed during specimen handling. Standard operating procedures and the good clinical laboratory practices were strictly adhered to.

### Laboratory Analysis

Tissue sections for ZN staining were prepared according to the site standard protocol. Briefly, the tissue samples were de-paraffinised twice by immersion in xylene-containing vessel, each for 3 minutes, followed by 10 dips immersion in 95%, 80%, and 70% ethyl alcohol. Thereafter, the tissue sections were flooded with carbol fuchsin on the staining rack, warmed until steam rose (not boiled), and left for 10 minutes to cool. Tissue sections were then washed well in distilled water and differentiated in 1% acid in 70% alcohol for 30 seconds. Then, 0.2% methylene blue was applied as counter stain for 1 minute. Stained slides were then blotted dry with Whatman filter paper and, thereafter, transferred to 3 changes of absolute alcohol and cleared in 3 changes of xylene. Finally, stained slides were mounted in distyrene plasticizer xylene (DPX) applied with cover slip.

### DNA Extraction Procedures

Extraction of mycobacterial DNA was performed by using 2 different commercial kits, QIAamp DNA FFPE Tissue Kit (Qiagen, Hilden, Germany) and prepIT•MAX (DNA Genotek, Ottawa, Canada). The Qiagen DNA extraction method was performed according to the manufacturer protocol while the prepIT•MAX DNA extraction protocol was modified accordingly to suite extraction of DNA from FFPE tissue specimen. Modifications included, but were not limited to, initial processing from de-paraffinisation to when tissue pellets were obtained before addition of MAX Buffers for DNA extraction. In brief, excess paraffin was trimmed off the sample tissue block and 8 tissue sections of 10 lm thick were cut. One mL xylene was added to the trimmed sections in a 1.5 microcentrifuge tube and then centrifuged at 14,000 rpm for 2 minutes at room temperature. Supernatant was pipetted out. One millilitre (mL) of absolute ethanol was added to the pellets and mixed by vortexing and later centrifugation at 14,000 rpm for 2 minutes and supernatant pipetted out. Thereafter, the 1.5 microcentrifuge tubes containing pellets were opened and incubated at 37°C for 10 minutes.

### Polymerase Chain Reaction for *rpo*B Gene

A set of primers – TR8, 5′-TGCACGTCGCGGACCTCCA-3′ and TR9, 5′-TCGCCGCGATCAAGGAGT-3′–was used to amplify the 157 base pairs (bp) segment of *M. tuberculosis* complex *rpoB* gene. In brief, 5 μl of template DNA was added to a final volume of 100 μl containing 10 μl of 10x PCR buffer, 6 μl of 1.5 mM MgCl_2_ (Promega), 8 μl of 10 mM dNTP's (SIGMA), 2 μl of 0.5 μM of each primer and 0.4 μl(2U) of Taq polymerase (Promega). The PCR conditions included 3 stages: initial denaturation at 95°C for 15 minutes (stage 1), followed by 45 cycles of denaturation at 94°C for 1 minute, annealing at 58°C for 1 minute, extension at 72°C for 1 minute (stage 2), and a final extension at 72°C for 10 minutes (stage 3). PCR was done on a programmable PTC-100 thermo cycler (Bio-Rad Laboratories, Foster City, CA, USA). The laboratory *M. tuberculosis* H37Rv standard strain was used as a positive control and DNA-free molecular grade (PCR) water as a negative control.

### Gel Electrophoresis

**FIGURE 1.**
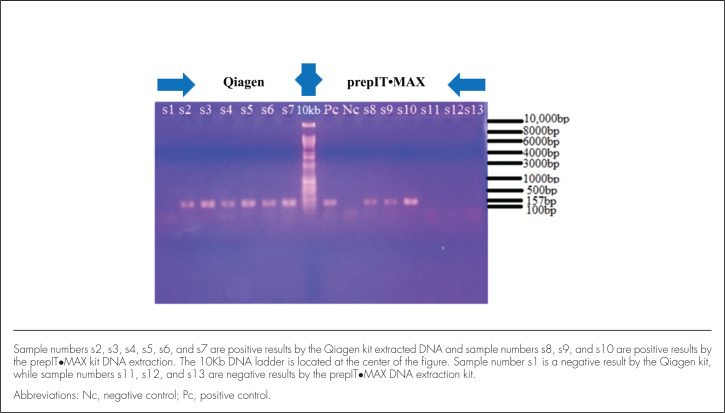
PCR Amplicons From Samples Extracted Using Both the Qiagen Kit and prepIT•MAX Kit

Gel electrophoresis was done at 80V for 2 hours on 1.5% aga-rose (Promega) in 1x TBE buffer and stained with ethidium bromide (conc 10 mg/mL) (SIGMA). The PCR amplicons were sized using 10 Kb-ladder. PCR amplicons were visualized under UV light and images electronically recorded and stored using a digital mobile phone camera (Samsung Galaxy S5, SM-G900H). The images were later transferred to the computer for interpretation. Expected band size of the target DNA fragment was 157 bp (**Figure 1**).

Sample numbers s2, s3, s4, s5, s6, and s7 are positive results by the Qiagen kit extracted DNA and sample numbers s8, s9, and s10 are positive results by the prepIT•MAX kit DNA extraction. The 10Kb DNA ladder is located at the center of the figure. Sample number s1 is a negative result by the Qiagen kit, while sample numbers s11, s12, and s13 are negative results by the prepIT•MAX DNA extraction kit.

Abbreviations: Nc, negative control; Pc, positive control.

### Data Management and Statistical Analysis

Data entry, cleaning and analysis was performed using IBM SPSS Statistics for Windows version 21.0 (IBM Corp., Armonk, NY, USA). The data collected included, TB infection as the dependent variable, and gender, site of biopsy, area of residence and microscopy histological features as independent variables. Descriptive statistics (Pearson *χ*^2^-test and Fisher's exact test) were performed to establish frequencies and association of factors where *p*-values less than .05 were considered statistically significant.

### Ethical Consideration

The institutional ethical clearance was obtained from MUHAS Institutional Review Board (IRB), Ref. No. MU/PGS/SAEC/Vol. XVI and permission to conduct research was obtained from MNH. To maintain confidentiality, laboratory and hospital patient identification numbers were assigned and used instead of names.

## RESULTS

Of the 160 archived tissue samples used in this study, 82 (51.2%) were collected from females and 78 (48.8%) were collected from males. The male-to-female ratio of study subjects was 1 to 1, of whom, 16 (10.0%) subjects were under 15 years, 106 (66.3%) between 15 and 45 years, and 38 (23.7%) over 45 years (**Table 1**). The mean age range was 33.00 ± 16.7 years. Specific body sites from which most samples were taken include: acetabulum, chest, heel, knee, perianal, peritoneum, pharynx, pleura, scalp, skin, spine, mandible, and lymph nodes.

**TABLE 1.** Distribution of Sampled Subjects By Gender and Age

Age Group	Male n (%)	Female n (%)	Total n (%)
<15	8 (5.0)	8 (5.0)	16 (10.0)
15 to 45	51 (31.9)	55 (34.4)	106 (66.3)
>45	19 (11.9)	19 (11.9)	38 (23.8)
Total	78 (48.8)	82 (51.3)	160 (100.0)

As stated earlier, DNA extraction was performed from each sample using 2 kits, prepIT•MAX and Qiagen, followed by PCR using extracts from both kits. With PCR results for samples extracted using the Qiagen kit, 12 (7.5%) out of 160 samples were found positive for *M. tuberculosis*, while 4 (33.3%) samples were found PCR positive with the prepIT•MAX kit. Four (33.3%) out of 12 PCR positive samples were positive with both kits and 8 (66.7%) PCR positive samples were detected from the Qiagen kit extraction alone. The 2 kits were significantly different in DNA extraction (*P*<.05) with the Qiagen kit being 3 times superior to the prepIT•MAX DNA extraction kit (**Table 2**).

**TABLE 2.** Qiagen and prepIT•MAX DNA Extraction Kits: Results By Proportions After Gel Electrophoresis of PCR Products*

	prepIT•MAX		
Qiagen	Negative n (%)	Positive n (%)	Total n (%)
Negative	148 (100.0)	0 (0.0)	148 (100.0)
Positive	8 (66.7)	4 (33.3)	12 (100.0)
Total	156 (97.5)	4 (2.5)	160 (100.0)

* Primers for rpoB gene were used to amplify the DNA target gene.

### Gel Electrophoresis Results

**FIGURE 2.**
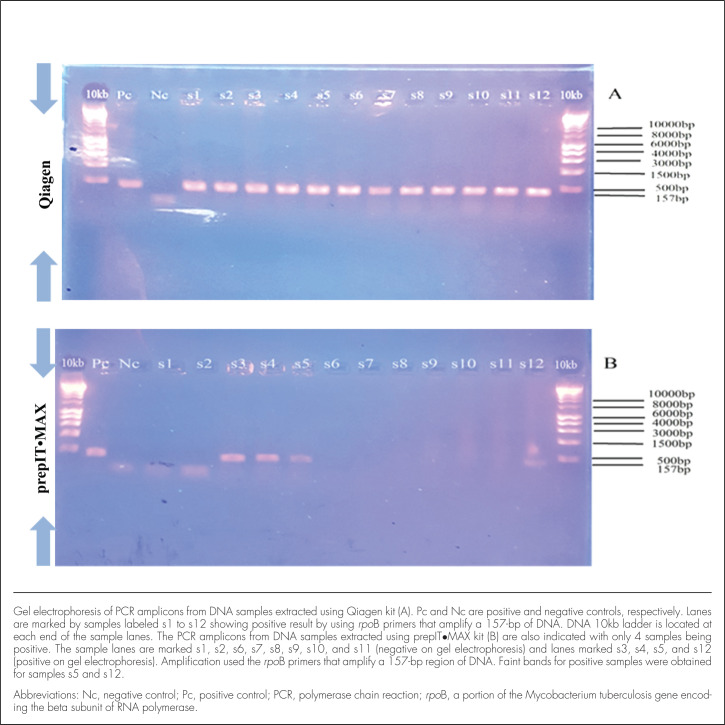
Gel Electrophoresis of PCR Amplicons From DNA Samples

The PCR amplicons obtained were run in 1.5% agarose gel; the results for both Qiagen and prepIT•MAX DNA extraction kits are shown in **Figure 1**. Comparable gel electrophoresis of PCR amplicons from DNA samples extracted using the Qiagen kit (A) and the prepIT•MAX kit (B) are also shown in **Figure 2**. In these figures, the clear difference between the two test kits is obvious: only 4 samples were extracted using the prepIT•MAX compared to 12 samples extracted using the Qiagen kit.

Gel electrophoresis of PCR amplicons from DNA samples extracted using Qiagen kit (A). Pc and Nc are positive and negative controls, respectively. Lanes are marked by samples labeled s1 to s12 showing positive result by using rpoB primers that amplify a 157-bp of DNA. DNA 10kb ladder is located at each end of the sample lanes. The PCR amplicons from DNA samples extracted using prepIT•MAX kit (B) are also indicated with only 4 samples being positive. The sample lanes are marked s1, s2, s6, s7, s8, s9, s10, and s11 (negative on gel electrophoresis) and lanes marked s3, s4, s5, and s12 (positive on gel electrophoresis). Amplification used the rpoB primers that amplify a 157-bp region of DNA. Faint bands for positive samples were obtained for samples s5 and s12.

Abbreviations: Nc, negative control; Pc, positive control; PCR, polymerase chain reaction; rpoB, a portion of the Mycobacterium tuberculosis gene encoding the beta subunit of RNA polymerase.

## DISCUSSION

This study focused on the evaluation of conventional versus molecular-based techniques for the identification of *M. tuberculosis* in FFPE tissues. No such study had previously been conducted in Tanzania, hence the proportion of cases detected based on the technique in the tissue samples is unclear. The value of archived FFPE tissue samples is in their ability to enable retrospective and prospective evaluation of large numbers of samples and track genetic changes over time in respect of infectious agents and genetic diseases.^2^ FFPE are suitable specimens that can provide sources of DNA from even deteriorated samples and can provide valuable information from small amounts of specimens within a short time.^3,4^ The 160 samples used in this study were collected over a period of 7 years.

Of 160 participants, 106 (66.3%) presumptive TB patients were 15 to 45 years of age suggesting that a larger proportion of relatively young to middle-age and active individuals reported as presumptive TB patients than any other age group. Similar findings were recently reported in the Serengeti ecosystem survey.^18,19^ The present study has shown that of 12 PCR positive samples, 8 (66.7%) were collected from patients aged 15 to 45 years. Available evidence reveals TB lymphadenitis to occur relatively early, after *M. tuberculosis* primary infection, frequently affecting young people in endemic countries.^20^ The female-to-male ratio of 1 to 1 obtained in this study is indicative of TB infection to be related to exposure rather than gender (**Table 1**).

This study obtained samples from different tissue sites. However, lymph node samples were the most common (n=127, 79.4%), constituting 10 (83.3%) out of 12 PCR positive specimens. This was similar to previous reports^21^ that used tissue samples from selected sites corresponding to TB infection. Lymph nodes are primary immune response sites prior to infection spread, and the first sites to show features suggestive of extrapulmonary TB. In children, however, the most serious forms are disseminated TB and TB meningitis. TB lymphadenitis is the most common form, accounting for up to 50.0% of extrapulmonary cases in children.^22^ Thus, finding a larger proportion of PCR positives in lymph nodes is not surprising.

The present study has shown superiority of PCR to detect TB in tissues previously reported as acid-fast bacillus (AFB) negative by ZN. Of the 160 FFPE tissue specimens tested, none were AFB ZN positive contrary to the previously reported 61.7% AFB positive.^20^ Our retrospective study used laboratory microscopic findings that are generally nonspecific with other conditions, such as sarcoidosis, syphilis, leprosy, Crohn's disease, rheumatoid arthritis, systemic lupus erythematous, and pneumoconiosis, having similar features.^23^ The failure of ZN to detect AFB in FFPE tissue specimen could be a result of paucibacillary nature of the tissue specimen.^24,25^ Presence of a smaller number of bacteria in tissue specimens might necessitate future bacterial culture plans to increase the DNA yield if similar studies are done. This can help to minimize false negative results that could be anticipated following direct extraction of mycobacterial DNA from FFPE tissues.

Twelve (7.5%) out of 160 tissue samples were PCR positive. Results from this molecular study, which used primers for *rpo*B gene segment in mycobacterial DNA, are important and highlight the superiority of PCR over conventional ZN-stain method. Similar results were reported in India, in a study that used insertion sequence IS6110 as a target gene for *M. tuberculosis* in tissues.^25^ Our study used FFPE tissues as DNA source. Such samples provide high-quality DNA with reported PCR success rates of 97%.^26^ In addition, others have reported that properly preserved and stored paraffin-embedded specimens can yield high-quality DNA given use of appropriate methods of extraction.^27^ Thus, we are confident that the PCR-positive samples confirmed TB cases in tissue specimens.

From this study, it is worthwhile to note the potential inability of the ZN stain to detect infection in paucibacillary tissues,^28,29^ 12 of which were PCR positive after DNA extraction using the Qiagen kit. The pathological features associated with such samples included well-formed granuloma (WFG), caseous necrosis (CN), and Langhans-type giant cells (LGC) (**Table 3**), signifying preferential localization of extrapulmonary TB lesions in these specific sites and their potential role of histopathology in TB diagnosis. Four (100%) of prepIT•MAX kit-extracted PCR-positive DNA samples had all of the main features (WFG, CN, and LGC). In general, 4 (33.3%) samples were detected by both Qiagen and prepIT•MAX extracted DNA while 8 (66.7%) were detected by Qiagen Kit extracted DNA alone. This reflects a 3 times superiority of Qiagen kit over the prepIT•MAX kit. Our findings provide clues of future complementary use of both Qiagen and prepIT•MAX kits in tissue samples to conventional ZN method. The prepIT•MAX kit has never been used in detection of mycobacterium DNA in FFPE, thus, the different outcomes might be attributed to the fact that the kit was not designated for that purpose.

**TABLE 3.** Association Between the Polymerase Chain Reaction and Microscopic Features Diagnosed Using Hematoxylin and Eosin Staining Technique

	Microscopic Features Diagnosed in H/E Staining Technique									
PCR Results	WFG	PFG	CN	LGC	WFG+CN	PFG+CN	WFG+CN+LGC	WFG+LGC	LGC+CN	Total
Qiagen										
Positive	1	0	0	1	0	0	10	0	0	12
Negative	2	4	1	5	4	1	121	9	1	148
Total	3	4	1	6	4	1	131	9	1	160
prepIT•MAX										
Positive	0	0	0	0	0	0	4	0	0	4
Negative	3	4	1	6	4	1	127	9	1	156
Total	3	4	1	6	4	1	131	9	1	160

Abbreviations: CN, caseous necrosis; H/E, hematoxylin and eosin; LGC, Langhans-type giant cells; PCR, polymerase chain reaction; PFG, poorly formed granuloma; WFG, well-formed granuloma.

Two studies previously conducted in India, compared the conventional diagnostic modalities of BACTEC culture and PCR test^30^ and IS6110-based PCR^31^ for detection of *M. tuberculosis* clinical samples of diversified nature and revealed an association between histological features – WFG, CN, and LGC (**Table 3**) – and molecular findings. Our study revealed a similar likelihood that *M. tuberculosis* presence in tissue samples might correlate with non-specific histopathological features. In endemic areas, diseases with chronic granulomatous lesions similar to extrapulmo-nary TB, such as sarcoidosis, syphilis, leprosy, Crohn's disease, rheumatoid arthritis, systemic lupus erythematosus, and pneumoconiosis, can be differentiated using molecular methods^23^ and, thus, indicate precise medical treatment.

Archived tissue samples are valuable source of biological material for epidemiologic research of the disease.^32^ DNA concentration may influence the sensitivity of PCR as suggested in previous studies,^21,33^ hence the need for an appropriate and optimized method of DNA extraction. Therefore, the extraction procedure should ensure effective lysis of mycobacteria and good recovery of the DNA from a complex mixture of tissue debris with minimal PCR inhibitors. Combined inhibitory factors that might interfere with MTB detection in archived samples are inevitable.^34^ This necessitates for dedicated control of inhibitory factors in order to suit molecular tools used for diagnosis. While the kits used in this study might not have the ability and level of quality for extracting of mycobacterial DNA from FFPE, we are sure these findings provide insights for valuable use of the kits in the future. A number of studies have addressed the problem of initial processing of mycobacterial samples and range from simple boiling and centrifugation^35^ to trapping of DNA on Chelex resin and bead-beating.^33^ It is important to observe these useful procedures for quality DNA.

So far, the study has shown a significant difference in performance between the 2 kits with p≤–.05. The kits comparatively differed in performance with reflection of the Qiagen kit 3 times superior to the prepIT•MAX. Previous studies clearly indicated a greater power of the Qiagen commercial kits in extracting genomic DNA with lysis reagents.^21,35^ The performance of prepIT•MAX in FFPE tissues is very promis ing, given that prepIT•MAX was used for the first time.

## CONCLUSION AND RECOMMENDATIONS

Based on the findings from this study, it is recommended that in a setting like the Tanzania national referral hospital, where a majority of tissue specimens are examined and archived, the PCR technique could be used for diagnosis of TB. The use of PCR will increase the detection rate and differentiate extrapulmonary TB from other chronic granulomatous diseases with similar lesions. In this study, a significant number of TB presumptive patients were found to be missed prior to the use of a molecular approach on archived tissues implying that under detection of extrapulmonary TB cases is not uncommon in African TB diagnostic settings. It is also important for clinicians to pinpoint exactly the most preferred specific tissue sites for *M. tuberculosis* in extrapulmonary TB cases. We further conclude that protocol optimization for prepIT•MAX kit is still needed for it to perfectly suit deploy ment as a tool for DNA extraction from tissues. Having this ‘dual purpose’ tool will particularly fit well in resource-limited countries by enabling diagnosis and screening of TB cases from both sputa and tissues where TB is clinically suspected. This preliminary information provides an insight and potential for future improvement of the kit for better results. Nevertheless, there is a great need for cost effectiveness studies as the technique might be important, but it is cost driven.

## References

[1] Wright DK, Manos MM. Sample preparation from paraffin-embedded tissues. In: Innis MA, Gelfand DH, Sninsky JJ, White TJ, eds. PCR Protocols: A Guide to Methods and Applications. San Diego, CA, USA: Academic Press; 1990: 153–158.

[2] Magdeldin S, Yamamoto T. Toward deciphering proteomes of formalin-fixed paraffin-embedded (FFPE) tissues. Proteomics. 2012;12(7):1045–1058. 10.1002/pmic.201100550. Medline 22318899 PMC3561704

[3] Impraim CC, Saiki RK, Erlich HA, Teplitz RL. Analysis of DNA extracted from formalin-fixed, paraffin-embedded tissues by enzymatic amplification and hybridization with sequence-specific oligonucleotides. Biochem Biophys Res Commun. 1987;142(3):710–716. 10.1016/0006-291X(87)91472-0. Medline 3548717

[4] Shibata DK, Arnheim N, Martin WJ. Detection of human papilloma virus in paraffin-embedded tissue using the polymerase chain reaction. J Exp Med. 1988;167(1):225–230. 10.1084/jem.167.1.225. Medline 2826637 PMC2188813

[5] Radomski N, Kreitmann L, McIntosh F, Behr MA. The critical role of DNA extraction for detection of mycobacteria in tissues. PLoS One. 2013;8(10):e78749. 10.1371/journal.pone.0078749. Medline 24194951 PMC3806855

[6] Godfrey TE, Kim SH, Chavira M, et al. Quantitative mRNA expression analysis from formalin-fixed, paraffin-embedded tissues using 5’ nuclease quantitative reverse transcription-polymerase chain reaction. J Mol Diagn. 2000;2(2):84–91. 10.1016/S1525-1578(10)60621-6. Medline 11272893 PMC1906896

[7] Musella V, Callari M, Di Buduo E, et al. Use of formalin-fixed paraffin-embedded samples for gene expression studies in breast cancer patients. PLoS One. 2015;10(4):e0123194. 10.1371/journal.pone.0123194. Medline 25844937 PMC4386823

[8] Guo T, Wang W, Rudnick PA, et al. Proteome analysis of microdissected formalin-fixed and paraffin-embedded tissue specimens. J Histochem Cytochem. 2007;55(7):763–772. 10.1369/jhc.7A7177.2007. Medline 17409379

[9] Scicchitano MS, Dalmas DA, Boyce RW, Thomas HC, Frazier KS. Protein extraction of formalin-fixed, paraffin-embedded tissue enables robust proteomic profiles by mass spectrometry. J Histochem Cytochem. 2009;57(9):849–860. 10.1369/jhc.2009.953497. Medline 19471015 PMC2728129

[10] Kokkat TJ, Patel MS, McGarvey D, LiVolsi VA, Baloch ZW. Archived formalin-fixed paraffin-embedded (FFPE) blocks: a valuable underexploited resource for extraction of DNA, RNA, and protein. Biopreserv Biobank. 2013;11(2):101–106. 10.1089/bio.2012.0052. Medline 24845430 PMC4077003

[11] Barrios-Payán J, Saqui-Salces M, Jeyanathan M, et al. Extrapulmonary locations of Mycobacterium tuberculosis DNA during latent infection. J Infect Dis. 2012;206(8):1194–1205. 10.1093/infdis/jis381. Medline 22732919

[12] Carruth L, Roess AA, Mekonnen YT, Melaku SK, Nichter M, Salman M. Zoonotic tuberculosis in Africa: challenges and ways forward. Lancet. 2016;388(10059):2460–2461. 10.1016/S0140-6736(16)32186-9. Medline 27871735 PMC7135042

[13] Johansen IS, Thomsen VØ, Forsgren A, Hansen BF, Lundgren B. Detection of Mycobacterium tuberculosis complex in formalin-fixed, paraffin-embedded tissue specimens with necrotizing granulomatous inflammation by strand displacement amplification. J Mol Diagn. 2004;6(3):231–235. 10.1016/S1525-1578(10)60515-6. Medline 15269300 PMC1867630

[14] Salian NV, Rish JA, Eisenach KD, Cave MD, Bates JH. Polymerase chain reaction to detect Mycobacterium tuberculosis in histologic specimens. Am J Respir Crit Care Med. 1998;158(4):1150–1155. 10.1164/ajrccm.158.4.9802034. Medline 9769274

[15] Park JS, Kang YA, Kwon SY, et al. Nested PCR in lung tissue for diagnosis of pulmonary tuberculosis. Eur Respir J. 2010;35(4):851–857. 10.1183/09031936.00067209. Medline 19741027

[16] Park DY, Kim JY, Choi KU, et al. Comparison of polymerase chain reaction with histopathologic features for diagnosis of tuberculosis in formalin-fixed, paraffin-embedded histologic specimens. Arch Pathol Lab Med. 2003;127(3):326–330. Medline 12653577 10.5858/2003-127-0326-COPCRW

[17] Shi SR, Cote RJ, Wu L, et al. DNA extraction from archival formalin-fixed, paraffin-embedded tissue sections based on the antigen retrieval principle: heating under the influence of pH. J Histochem Cytochem. 2002;50(8):1005–1011. 10.1177/002215540205000802. Medline 12133903

[18] Mbugi EV, Katale BZ, Lupindu AM, et al. Tuberculosis infection: occurrence and risk factors in presumptive tuberculosis patients of the Serengeti ecosystem in Tanzania. East Afr Health Res J. 2017;1(1):19–30. 10.24248/EAHRJ-D-16-00319 PMC8279301 34308155

[19] Mbugi EV, Katale BZ, Streicher EM, et al. Mapping of Mycobacterium tuberculosis complex genetic diversity profiles in Tanzania and other African countries. PLoS One. 2016;11(5):e0154571. 10.1371/journal.pone.0154571. Medline 27149626 PMC4858144

[20] Eshete A, Zeyinudin A, Ali S, Abera S, Mohammed M. M. tuberculosis in lymph node biopsy paraffin-embedded sections. Tuberc Res Treat. 2011;2011:127817. 10.1155/2011/127817 22567262 PMC3335535

[21] Chawla K, Gupta S, Mukhopadhyay C, Rao PS, Bhat SS. PCR for M. tuberculosis in tissue samples. J Infect Dev Ctries. 2009;3(2):83–87. Medline 19755735 10.3855/jidc.53

[22] Negi SS, Anand R, Pasha ST, et al. Detection of M. tuberculosis in clinical samples of diversified nature by IS6110 based PCR. J Commun Dis. 2006;38(4):325–332. Medline 17913208

[23] Asano S. Granulomatous lymphadenitis. J Clin Exp Hematop. 2012;52(1):1–16. 10.3960/jslrt.52.1. Medline 22706525

[24] Alvarado-Esquivel C, García-Corral N, Carrero-Dominguez D, et al. Molecular analysis of Mycobacterium isolates from extrapulmonary specimens obtained from patients in Mexico. BMC Clin Pathol. 2009;9:1. 10.1186/1472-6890-9-1. Medline 19272158 PMC2660362

[25] Taylor GM, Worth DR, Palmer S, Jahans K, Hewinson RG. Rapid detection of Mycobacterium bovis DNA in cattle lymph nodes with visible lesions using PCR. BMC Vet Res. 2007;3:12. 10.1186/1746-6148-3-12. Medline 17567891 PMC1904440

[26] Lin J, Kennedy SH, Svarovsky T, et al. High-quality genomic DNA extraction from formalin-fixed and paraffin-embedded samples deparaffinized using mineral oil. Anal Biochem. 2009;395(2):265–267. 10.1016/j.ab.2009.08.016. Medline 19698695 PMC2764035

[27] Pikor LA, Enfield KSS, Cameron H, Lam WL. DNA extraction from paraffin embedded material for genetic and epigenetic analyses. J Vis Exp. 2011;(49):2763. 21490570 10.3791/2763 PMC3197328

[28] Jin XJ, Kim JM, Kim HK, et al. Histopathology and TB-PCR kit analysis in differentiating the diagnosis of intestinal tuberculosis and Crohn's disease. World J Gastroenterol. 2010;16(20):2496–2503. 10.3748/wjg.v16.i20.2496. Medline 20503449 PMC2877179

[29] Peter JG, Theron G, Muchinga TE, Govender U, Dheda K. The diagnostic accuracy of urine-based Xpert MTB/RIF in HIV-infected hospitalized patients who are smear-negative or sputum scarce. PLoS One. 2012;7(7):e39966. 10.1371/journal.pone.0039966. Medline 22815718 PMC3392260

[30] Negi SS, Khan SFB, Gupta S, Pasha ST, Khare S, Lal S. Comparison of the conventional diagnostic modalities, bactec culture and polymerase chain reaction test for diagnosis of tuberculosis. Indian J Med Microbiol. 2005;23(1):29–33. 10.4103/0255-0857.13869. Medline 15928418

[31] Afghani B, Stutman HR. Polymerase chain reaction for diagnosis of M. tuberculosis: comparison of simple boiling and a conventional method for DNA extraction. Biochem Mol Med. 1996;57(1):14–18. 10.1006/bmme.1996.0003. Medline 8812720

[32] Hens K, Nys H, Cassiman J-J, Dierickx K. Biological sample collections from minors for genetic research: a systematic review of guidelines and position papers. Eur J Hum Genet. 2009;17(8):979–990. 10.1038/ejhg.2009.9 19223929 PMC2986563

[33] Tell LA, Leutenegger CM, Larsen RS, et al. Real-time polymerase chain reaction testing for the detection of Mycobacterium genavense and Mycobacterium avium complex species in avian samples. Avian Dis. 2003;47(4):1406–1415. 10.1637/7063. Medline 14708989

[34] Barcelos D, Franco MF, Leão SC. Effects of tissue handling and processing steps on PCR for detection of Mycobacterium tuberculosis in formalin-fixed paraffin-embedded samples. Rev Inst Med Trop Sao Paulo. 2008;50(6):321–326. 10.1590/S0036-46652008000600002. Medline 19082372

[35] Aldous WK, Pounder JI, Cloud JL, Woods GL. Comparison of six methods of extracting Mycobacterium tuberculosis DNA from processed sputum for testing by quantitative real-time PCR. J Clin Microbiol. 2005;43(5):2471–2473. 10.1128/JCM.43.5.2471-2473.2005. Medline 15872286 PMC1153782

